# Feasibility of a Novel Socially Assistive Robot With Smart Sensing to Care for Individuals With ADRD

**DOI:** 10.1093/geroni/igaf122.1542

**Published:** 2025-12-31

**Authors:** Sajay Arthanat

**Affiliations:** University of New Hampshire, Durham, New Hampshire, United States

## Abstract

Several studies have highlighted the scope of socially assistive robots (SARs) to care of individuals with ADRD in supervised or institutionalized settings. However, the feasibility and effectiveness of SARs to independently support aging-in-place at homes and retirement facilities are yet to be explored. This overview section will highlight the significance of our project to support caregiving and daily living routines of individuals with ADRD. We will elucidate the technological framework used to develop a novel SAR, Mobile Assistive Robot with Smart Sensing (MARSS), with multi-modal capabilities to deliver care protocols. We will present findings from an ongoing mixed-method pilot study with five caregiver-care recipient dyads who utilized MARSS in the community for 3-6 months. Findings pertaining to feasibility, preliminary effectiveness and acceptance of the SAR will be presented through analysis of in-depth qualitative data (pre-and post-intervention) as well as monthly data gathered using Goal Attainment Scaling. Qualitative content analysis of data gathered through semi-structured interviews (based on the Unified Theory of Acceptance and Use of Technology) prior to the introduction, during usage, and end of trial is currently underway. The Goal Attainment Scaling involves individualized care goals set with the caregiver that considers the degree of accomplishment of each goal, importance of each goal (weight), and the difficulty with the goal at baseline. The project employs the NIH Stage Model for Intervention Development to identify and validate the target behaviors among care partners leading to potential adoption of MARSS, and to iteratively refine the technology towards a randomized controlled trial.

